# Use of iron mine tailing as fillers to polyethylene

**DOI:** 10.1038/s41598-021-86456-z

**Published:** 2021-03-29

**Authors:** Ítalo R. Coura, Ottavio R. D. R. Carmignano, Ana Pacheli Heitmann, Fernando S. Lameiras, Rochel M. Lago, Patrícia S. de O. Patricio

**Affiliations:** 1 Department of Chemistry, Centro Federal de Educação Tecnológica de Minas Gerais, CEFET-MG, Av. Amazonas 5253, Belo Horizonte, MG 30421-169 Brazil; 2 Department of Chemistry, Universidade Federal de Minas Gerais, Av. Antônio Carlos 6627, Campus Pampulha, Belo Horizonte, MG 31270-901 Brazil; 3 Centro de Desenvolvimento de Tecnologia Nuclear, Av. Antônio Carlos 6627, Campus Pampulha, Belo Horizonte, MG 31270-901 Brazil

**Keywords:** Environmental sciences, Materials science

## Abstract

The iron mine tailings accumulation in dams is an environmental and economic problem. The composite based on high-density polyethylene/iron mine tailing production for the application of wood plastic and some items of domestic plastic industry can be a good alternative to reduce the rejects in the environment. This work presents the influence of the processing methodology in the mechanical, thermal and morphological properties of composites based on the high-density polyethylene/iron mine tailing. Four methodology processing by continuous and/or batch mixing were available. The iron mine tailing particles in the polymer matrix promoted an increase in mechanical strength and thermal stability. Besides, the particles acted as flame retardant. The iron mine tailing materials produced using batch mixing showed more significant modifications in the properties due to the better dispersion of the filler as shown by scanning electron microscopy.

## Introduction

The accumulation of iron mine tailing is an environmental problem that has been much discussed in academia since the 1970s, mainly due to the high amount annually generated worldwide. The global production of solid waste in the mining industry was estimated at approximately 14 billion tons in 2010^1^ and is constantly growing.

Among the mining tailings, the bauxite (Al_2_O_3_, red mud)^2, 3^ and iron mine tailing^4^ can be highlighted. In Brazil, the accumulation of iron mine tailing in dams gained prominence after accidents involving hundreds of victims, causing huge environmental, economic and social damage to the country. In general, the sources mined by iron mine tailing may have several mineral structures, such as hematite, goethite and limonite; all composed of iron (III) oxide^5^.

There is a research utilizing this waste for useful applications minimizing environmental damage and generating higher value added products^6–8^. A viable alternative is the usage as a filler, for polymers preferably, if combined with the use as a functional filler. From the point of view of reuse, the main advantage is the consumption of significant quantities of byproducts of the mining industry.

Inorganic particles are highly used to reduce polymer costs (filler) and to produce improved performance materials (functional charge)^9–11^. Commercially, the use of calcium carbonate, kaolin, mica, quartz and talc can be highlighted as filler. Some of the expected functions of fillers are changes in the mechanical properties, improvements in the surface finish, changes in durability, electrical resistance, increasing in dimensional stability, etc.

In the scientific literature, studies are proposed to investigate the influence of additive-related parameters on polymer properties, such as: size, size distribution and content of inorganic particles dispersion and distribution of charges in the polymeric matrix, surface tension, surface adhesion polymer/particle, among others^12^. The relationship between the particles content added in composites and polymer property modifications is reported in the literature for various types of polymer composites.

Among the mining industry tailings and by-products described in the literature acting as polymer additives are: (1) bauxite mining by-products (Al_2_O_3_, red mud)^2, 3^, (2) iron^4^, (3) tailings from tungsten-based mineral mining^13^, (4) limestone mines^14^ and (5) phyllosilicate mines (Muscovites)^12^.

The involved polymers evaluated with the additives are polymethyl methacrylate (PMMA) and polyvinyl chloride (PVC)^2^, polypropylene (PP)^4^, polyester resins^14^, high-density polyethylene (HDPE) and epoxy resin^3, 12^. In these examples, the polymers generally have the highest content among the composition of the composites.

In their research, Souza^15^ produced various polymer composites, employing mining waste as reinforcing fillers in polymeric matrices from recycled plastics, including HDPE. An important conclusion by the author was to determine the limiting concentration of tailings in the polymeric matrix. The material could hold up to 25% by mass of mining waste with improvements in mechanical properties and durability; over 80, even in outdoor areas. Onitiri and Akinlabi^4^ used 20% by volume of iron ore tailings as maximum amount in the investigated composites.

HDPE is a polymer produced and consumed in large quantities worldwide. As a commodity plastic, it is mainly used in everyday packaging and utensils^16^. Due to the large amount discarded, coupled with the longtime of decomposition in the environment, there is a large interest in the use of this post-consumer polymer, or the manufacturing of utensils with longer service life. As a HDPE is a thermoplastic polymer mechanical processing can be performed facilitating the production of matrix/inorganic filler composites without the need for major modifications in the processing equipment. Composites based on HDPE as matrices can substitute the pure HDPE in the production of plastic wood^17^, the same items of domestic plastic industry and other products as pallets, tables, etc.

The objective of this work is to evaluate the use of iron mine tailing as filler for HDPE. Methodologies involving mechanical processing with batching equipment, continuous production and a combination of both were used for the preparation of composites with HDPE and 20%wt iron mine tailing. The products obtained were investigated in relation to their physicochemical, mechanical, structural, thermal and morphological properties.

## Experimental

### Material

The HDPE used from Braskem designated by IE59U3 has a flow index of 5 g/10 min at 190 °C with 2.16 kg weight. The samples were collected in the pond as tailings from an iron ore concentrator. Previous work had related the characterization of the iron mine tailing. The chemical composition of the sample is Fe_3_O_4_/Fe_2_O_3_/FeO(OH), SiO_2_, and Al_2_O_3_. The iron oxides identified by X-ray diffraction were hematite, martite, magnetite, and goethite; and the detected gangue minerals were quartz, gibbsite, and kaolinite^18^.

### Methods

HDPE/iron mine tailing composites were produced by thermal processing using different methodologies but the same content of inorganic particles (20%wt). The E1 sample was produced using the single screw extruder from the Thermo Scientific, Rheomex 19/25 QC model. HDPE and iron mine tailing alternating layers were introduced in the feeding hopper and submitted to the extrusion processing. The conditions of processing were 180 °C in the three heat zones and at 20 rpm. Then the material produced was pelletized and designated E1. Afterwards, a new processing cycle was initiated and E2 samples were obtained. The E1 pellets were introduced back into the extruder and processed under the same conditions. The sample D20 was prepared in the homogenizer M. H. Equipment, model MH-100. The mix was maintained in the chamber for 1.5 min under maximum rotation. The composite was granulated in a knife mill KIE, model MAC250BX. Finally, the ED composite was prepared in the homogenizer in the same conditions used in the preparation of D20 composite. The 50%wt polymer/iron mine tailing and master batch were mixed, then pelleted. The pellets ED and HDPE were put in the alternating layers in the feeding hopper and submitted the extrusion process in the content calculated to be diluted to 20%wt of the iron mine tailing. The very same processing condition described in the preparation of the composites E1 and E2 was employed in the preparation of ED composite.

The sample D20 was prepared in the homogenizer M. H. Equipment, model MH-100. The mix was maintained in the chamber for 1.5 min under maximum rotation. The composite was granulated in a knife mill KIE, model MAC250BX. Finally, the DE composite was prepared mix 50%wt polymer/iron mine tailing, master batch, that after granulated was put into the extruder for dilution until 20%wt iron mine tailing. The similar condition was employed in the preparation.

All samples were pressed as sheets with an approximate size of 150 × 100 × 1 mm, on a hydraulic press with heating of SOLAB brand, model SL11 up 10 MPa. The pellets were introduced in a mold pressing during 1 min at 150 °C.

Composites samples were put in a crucible bowl and weighed. The heating rate was 10 °C/min up until 600 °C. The samples were maintained in the chamber for 4 h at 600 °C. The residual mass was weighed.

Thermogravimetric (TG) measurements were performed on a SHIMADZU TG/DTA 60H thermal analyzer, with samples weighing about 7 mg at ambient temperatures up to 900 °C at 10 °C min^−1^ under a N_2_ atmosphere. DSC curves were performed on DSC-60 Shimadzu thermo analyzer. Samples were put onto alumina pans, and after that they were hermetically sealed. Two runs were evaluated: heating in the temperature range from 30 to 180 °C, maintaining at final temperature for 3 min, cooling to − 30 °C and heating again to 100 °C at a rate of 10 °C min^−1^ under a dynamic nitrogen atmosphere (50 mL min^−1^). The crystallinity index ($Xc$) of the polymers and the composites was calculated from Eq. (1):

1 $$Xc = \frac{{\Delta H_{n} \times 100}}{{\Delta H_{0} \times w\,(polymer)}}$$

where *ΔH*_0_ (J g^−1^) is the melting enthalpy of the sample as the heat of fusion for 100% crystalline polymers, $\Delta H_{n}$ (J g^−1^) is the melting enthalpy calculated from DSC curve and *w* is the weight fraction of the polymer’s samples^19^.

Infrared spectra were obtained on Shimadzu Prestige 21 spectrophotometer equipped with an attenuated total reflection accessory (FTIR-ATR with Krs-5 crystal). The spectra were collected in the range of 4000 to 400 cm^−1^ with a scan number of 60 and resolution of 4 cm^−1^.

Three samples of each nanocomposites were evaluated by FTIR. The spectra have been normalized by the intensity of the absorption band 1473 cm^−1^.

The morphological studies were performed by scanning electron microscopy and X-ray dispersive energy spectrometry (SEM; Shimadzu, model SSX-550). The samples were covered with gold for the tests used the sputtering Quick Coater Sanyu Electron SC-701. Images were collected using an electron beam with an acceleration voltage equal to 10 kV. Samples’ fracture regions were investigated by SEM.

Uniaxial tensile tests were performed on a universal testing machine Shimadzu Universal, model Autograph AG–X. To each type of material 8 samples with dimensions at 40 mm to initial length and a cross-sectional area of 6 mm^2^ (1 × 6 mm) were performed. The speed of the test was 110 mm/min. The values of mechanical properties corresponded to the arithmetic average of the measurements. The means and superior variances for the values of Young’s modulus and tensile strength were evaluated by *t*-Test and F-Test statistics with a 95% confidence level. Statistical analyses were performed using Microsoft Excel®.

The vertical flammability test was performed based on UL (“Underwriter’s Laboratory”) 94 specification. HDPE and E1 samples were subjected to the flammability test. For the test, 5 samples from each sample were obtained with dimensions of 144 mm in height; 13 mm wide; 3 mm thick.

## Results

**Figure 1:**
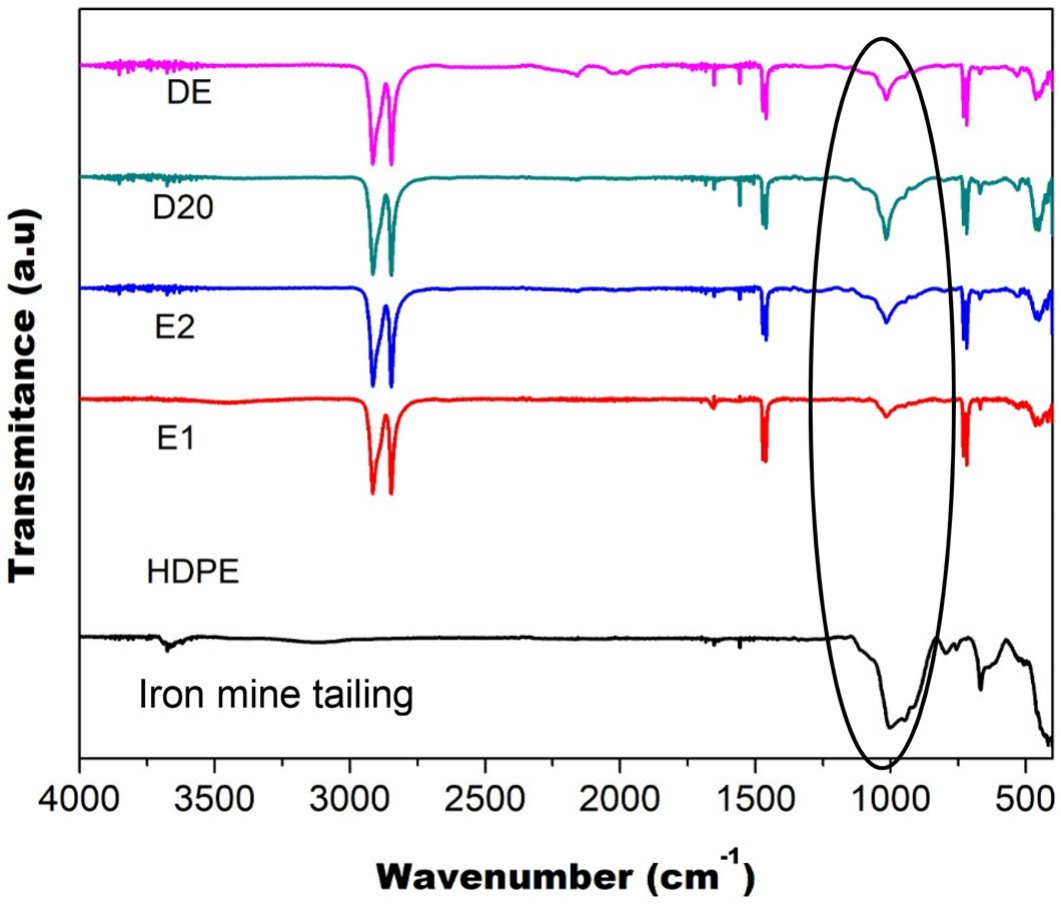
FTIR spectra of the HDPE, iron mine tailing and composites.

Figure 1 shows the HDPE, iron mine tailing and composites FTIR spectra and Table 1 presents spectra band assignment for HDPE and iron mine tailing. The two more intense bands identified in the FTIR spectrum of the HDPE appear at 2900 and 700 cm^−1^ associated with stretching vibrations and C–H bond deformations, respectively. In the iron mine tailing spectrum, absorption bands were observed approximately 1000 and 450 cm^−1^. These bands can be attributed to the Si–O–Si^20^ and Fe–O vibrations^21^ as highlighted by the circle shown in Fig. 1. In the spectrum of composite materials, the presence of the characteristic bands of HDPE spectra and iron mine tailing was identified.

**Table 1:** FTIR frequency wavenumber (cm^−1^) and its assignment of samples.

FTIR wavenumber (cm^−1^)	Band assignment	Samples
3000–2760	νCH_2_	HDPE
2080–1900	CH_2_ bands	HDPE
1510–1410	δCH_2_	HDPE
1150–820	Si–O–Si	Iron mine tailing
780–670	ρ[CH_2_]_n_	HDPE
550–400	Fe–O	Iron mine tailing

The main difference found between the composite’s spectra is the intensity of the vibration-related bands present in the iron mine tailing (inorganic particles). The Si–O–Si group band was evaluated, with a wavenumber in the range between 1200 and 800 cm^−1^, comparing the areas presented by the composite material spectra.

FTIR spectra were normalized to allow semi-quantitative analysis from the intensity of Si–O–Si bands of each composite. The area related to the absorption band in sample E1 is smaller than the others, followed by E2 and DE, with sample D20 having the highest value. The area of the bands was related to the content of iron mine tailing in the composites. The larger area is related to the higher iron mine tailing content in the samples. The samples prepared by homogenizer (D20 and DE) have higher iron mine tailing content than composites prepared in the extruder (E1 and E2), either by one or two processing cycles. During extrusion of the composite there is loss of filler, in specific, the iron mine tailing, in the feed and barrel extruder has led to the change in content in the composite**.**

The use of FTIR spectra to evaluate the proportion of iron mine tailing present in the composite is very interesting. The technique presents as advantages the agility in the preparation of the samples since it does not demand specific preparation, allied to the need of small amount of sampling. In general, the higher availability of this equipment and the lower cost of analysis can also be highlighted. In order to support these results, the iron mine tailing content was evaluated by thermogravimetric analysis and gravimetric analysis using a muffle furnace.

**Figure 2:**
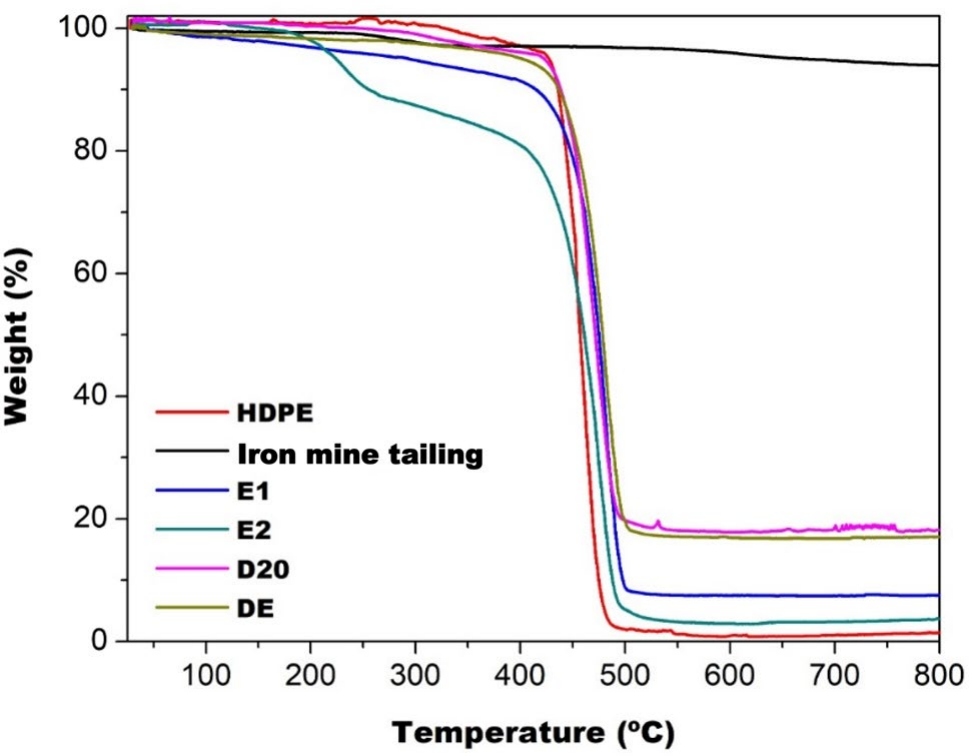
TGA curves of HDPE, iron mine tailing and polymer composites.

Figure 2 shows the thermogravimetric curves obtained from pure HDPE and composites. Pure HDPE presented an event related to thermal degradation, with mass loss in a single step, which occurs from 444 to 482 °C. According to the literature, degradation occurs due to the homogeneous structure of HDPE that decomposes into ethylene monomers^22^. After the temperature of 600 °C, the loss was 98% of its total mass.

The thermal degradation curve obtained from the inorganic material showed loss of approximately 1.2% of mass, in the range of 273 to 320 °C. The total mass loss after 600 °C was approximately 4.0%. It is well known that these oxides are stable at this temperature and do not thermally degrade. These losses are related to the presence of hydration water, which is retained in the oxide structure, being released at higher temperatures, followed by water even more strongly linked to the oxide structure^23^. In addition, events involving silicates, possibly dehydration, should be considered^6^.

**Figure 3:**
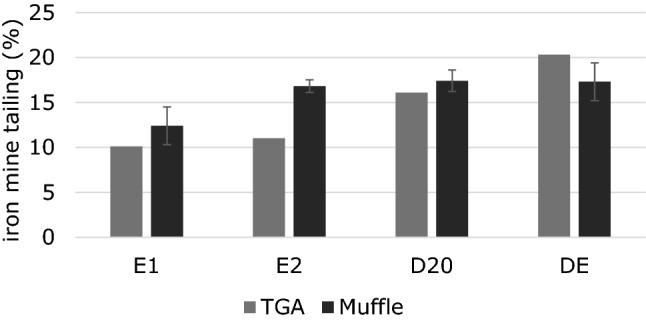
Comparison between TGA results and thermal decomposition for composites.

The thermal event, which occurred at lower temperatures in the composites TG curves, was associated with two types of events that may occur concurrently or isolated: (1) that occurred in the TGA curves of iron mine tailing, related to the release of water trapped in the oxide structure and/or (2) degradation and chains of smaller sized polymers due to thermal degradation promoted during processing. For the TGA curves of the composites it was observed a mass loss greater than the maximum expected value for the water content trapped in the oxide structure. The residual mass analysis of the samples, shown in Fig. 2, demonstrated that the samples processed in the homogenizer, D20 and DE, presented values of 16% and 20% respectively. For the samples produced only by extrusion process, E1 and E2, the values obtained were less than 10 and 11%. In the case of samples prepared by extrusion, the inorganic particle was retained in the equipment compartments. The results obtained by thermogravimetric analysis were compared with those conducted in the muffle furnace, as shown in Fig. 3.

The residual masses determined for samples E1, E2, D20 and DE were, respectively, 12.4, 16.8, 17.4, 17.3%. The results showed differences, but both indicated that the materials produced using the homogenizer show low loss of inorganic particles during processing. In addition, the mass loss of the residue when the composite is processed by extruder only is evident, regardless of the method evaluated.

**Figure 4:**
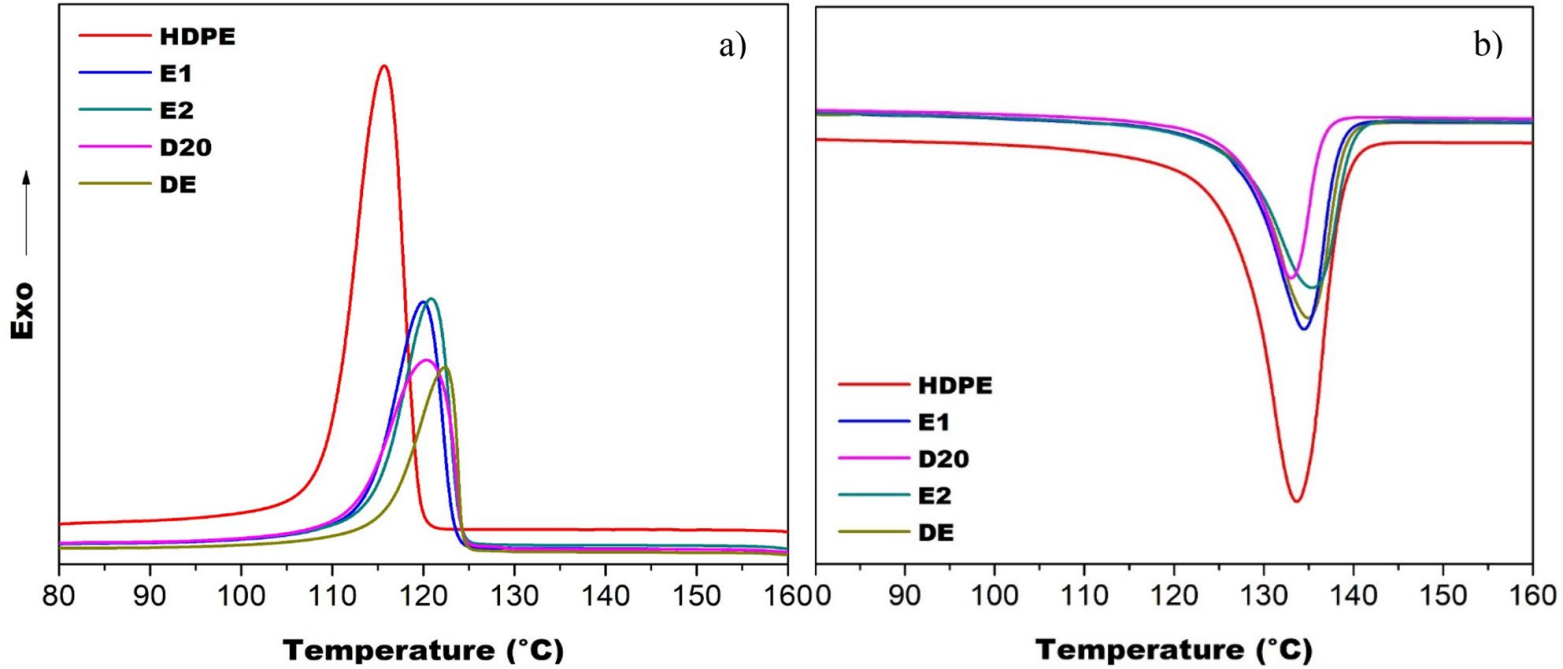
DSC curves during (**a**) second heating and (**b**) cooling of the composite materials and pure HDPE.

Figure 4 shows the DSC curves obtained during heating (a) and cooling (b) of the composites for crystallization analysis. The data obtained are described in Table 2, which are: crystalline melting temperature (Tm), crystallization temperature (Tc) and degree of crystallinity (Xc) obtained by the DSC curves. The crystallinity calculation was performed by Eq. (1).

**Table 2:** Properties obtained by DSC analysis on the second heating and on the cooling of the composites.

Samples	T_m_ (°C)	Tc (°C)	X_c_ (%)
HDPE	134	116	69.0
E1	134	120	71.4
E2	135	121	66.6
D20	136	120	64.3
DE	133	122	62.6

Analyzing the results obtained for HDPE-based composites and iron mine tailing, it was observed that the addition of inorganic particles did not significantly change the crystalline melting temperature of the matrix. However, it was also observed that the crystallization temperature increased for all composites when compared to pure polymer. This result can be justified by the presence of tailings acting as a nucleating agent, providing the fastest crystallization from the melt. Smaller crystals are formed, although the crystal structure is preserved^24^. Moreover, the tendency of a decrease in the crystallinity index corroborates as a nucleating agent of the fillers.

The results of the mechanical properties are in the Table 3. It was observed that the presence of inorganic particles from the iron mine tailing promoted an increase of Young’s modulus of the HDPE by up to 41%. The composites that passed through the homogenization equipment presented the highest increases. Evaluating the tensile strength, the materials were statistically divided into two groups: (1) HDPE, E1, E2 and (2) DE, D20. The composites prepared using the homogenizer presented a tensile strength increase close to 15%. For those obtained using extruder it was not observed a significant change.

**Table 3:** Properties of mechanical tests of HDPE and HDPE/iron mine tailing composites.

Samples	Young’s Modulus (MPa)	Tensile strength (MPa)
HDPE	778.2 (± 57.0)	34.2 (± 1.4)
E1	960.9 (± 32.8)	34.6 (± 0.9)
E2	940.1 (± 44.2)	33.4 (± 1.3)
D20	1098.5 (± 41.1)	38.3 (± 1.8)
DE	1044.0 (± 47.1)	37.3 (± 2.7)

Gungor^25^ produced HDPE-based composite and metallic Fe powder. The presence of iron particles promoted a gradual decrease of the tensile strength limit with increasing the proportion of fillers up to 18%. However, there was also a gradual increase in the elastic modulus of composites in relation to pure polymer of up to 48%. Unlike the results presented by the author, the addition of iron mine tailing improved both tensile strength and Young's modulus.

The results showed that inorganic particles from iron mine tailing have a filling function (since, as unwanted tailings in the industry, they have a low sales value), reinforcement (because it significantly increases mechanical resistances for a filler with Fe), and act as nucleation agent.

**Figure 5:**
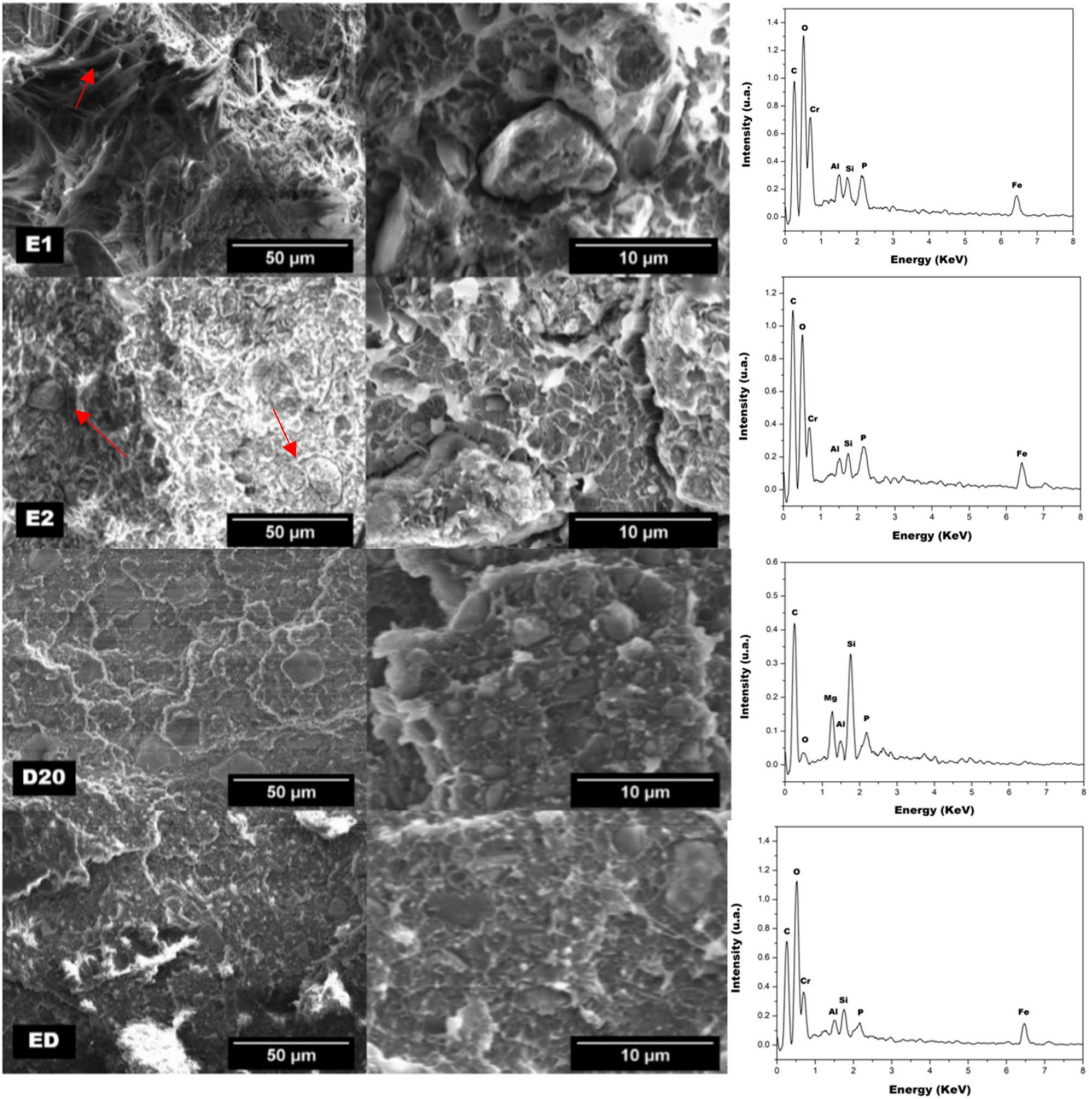
SEM images of HDPE/iron mine tailing composite materials and EDS spectra of films (right). The arrows highlight the filaments observed in the SEM image of the composite E1 and particles of iron mine tailing in the SEM image of composite E2.

After uniaxial tensile tests, SEM images of the samples were performed. The HDPE/iron mine tailing composites images and their respective EDS graphics are shown in Fig. 5.

It was noted in the fractures images of material E1, the formation of filaments (shown by the arrow), indicating a plastic deformation before rupture, common for pure HDPE. In the EDS graph, it was possible to confirm the presence of the fillers within the composite, without formation of large agglomerates (approximately 10 µm).

Gungor^25^ produced composites based on HDPE and Fe powder. The results showed poor fillers/matrix interaction characterized by the presence of interfacial vacancies. The author observes that the fracture presents matrix stretching, characteristic of ductile materials and particle agglomeration, characterized by the size difference between the particles. In the present work, the presence of stretching in the matrix was observed only in the sample E1, related to the lower proportion of fillers in the matrix, which justifies the less intense modification of the composite in relation to the pure polymer.

The fractures observed for composites E2, D20 and DE were identified as characteristics of fragile materials, which present less plastic deformation until rupture. Particle sizes were larger in E2 than in the other composites (shown by the arrow). This indicates the formation of clusters. In materials D20 and DE, no agglomerates were observed in this magnitude. This indicates better filler dispersion by batch mixer processing, since these materials have the same amount of fillers as E2. Some inorganic particles of materials produced using homogenizer presented submicron sized, indicating that processing may have led to a reduction in their particle size. The better dispersion of filler in the matrix in the composite prepared using the homogenizer can justify the higher reduction of the X_c_, obtained by DSC curve, since that the action of the particles as a nucleating agent is more efficient.

The EDS spectra confirmed that the observed phase is composed of metal elements and HDPE-related carbon atoms. The presence of Fe, P, Si, Al and Cr elements are characteristic of iron mine tailing, as described by Segura et al.^19^.

**Figure 6:**
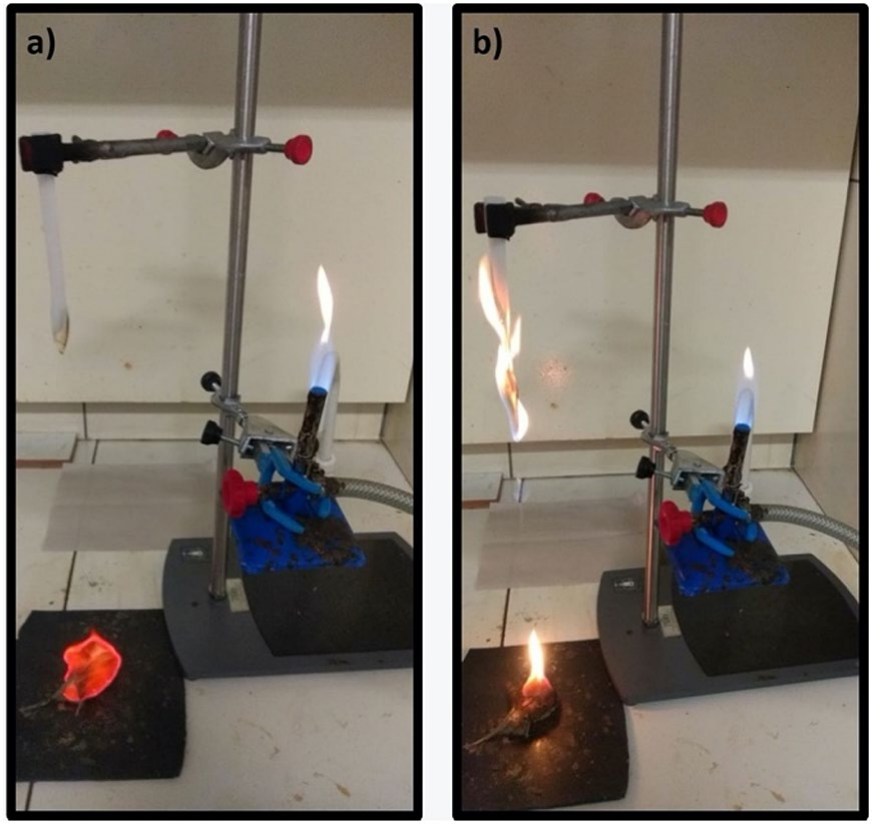
HDPE flammability test: (**a**) first application of flame for 10 s, (**b**) self-sustained flame burning in polymer.

**Figure 7:**
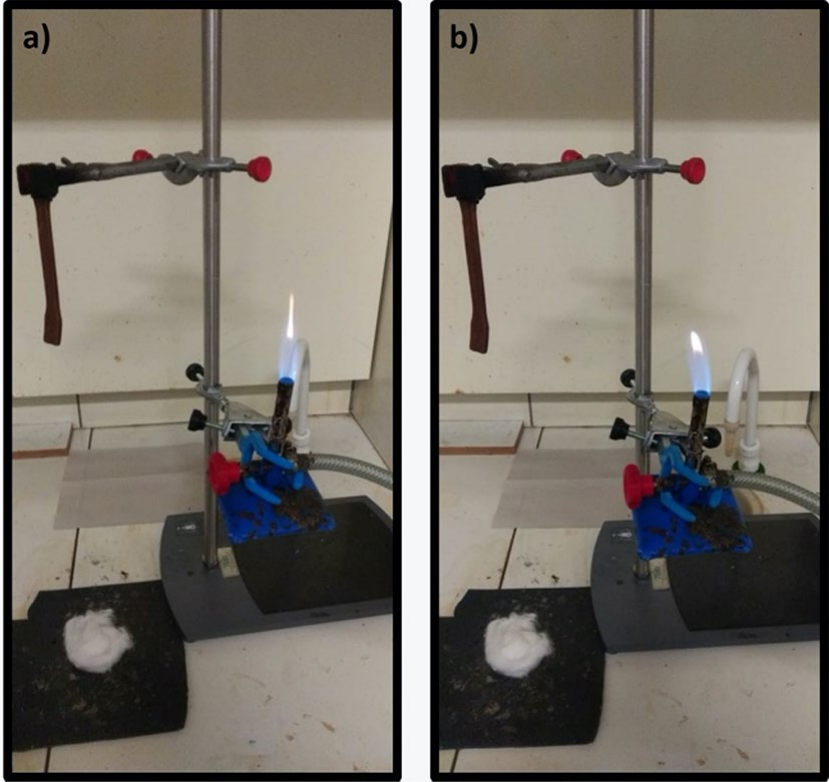
E1 flammability test: (**a**) first application of flame for 10 s, (**b**) self-sustained flame burning in polymer.

The flammability test was performed to evaluate if the iron mine tailing can act as flame retardant of the polymer during burning. This test evaluates the material's ability to propagate the flame in the same direction as pyrolysis products propagate, the vertical direction. For this reason, this is a very rigorous test as burning conditions are purposely favorable. Figures 6 and 7 shows the images of the samples during the flammability test.

When the samples first met the flame for 10 s, the flame was extinguished, although the polymer dripped onto the cotton, as shown in Fig. 6a. In Fig. 7b, after ignition and removal of the initial flame, the pure HDPE samples did not promote flame extinction within 50 s. Manual extinction was necessary to avoid consuming the entire specimen. For this reason, it was not possible to classify HDPE according to UL 94.

The samples E1 showed self-extinguishing of the flame at the moment of removal of the sample from the initial flame. This behavior was observed for both times when the specimen was led to the flame as shown in Fig. 7a, b. The samples showed no incandescence and no dripping occurred. With the results presented, samples of composite E1 were classified as V_0_ according to UL 94. According to UL 94, the material can be classified as V_0_ when the sum of the flame duration times is not more than 50 s and the cotton layer does not ignite by dripping the material. Therefore, this result indicates that iron mine tailing can act effectively as a flame retardant, preventing the burning of HDPE.

Krehula et al.^26^ studied HDPE-based composites and pine particles such as wood plastic composites (WPC) using flame retardant additives (ammonium polyphosphate and pentaerythritol) and reinforcing fillers (CaCO_3_ and SiO_2_). The authors state that mixing the additives resulted in higher flame retardancy, the presence of SiO_2_ increased this capacity due to reactions between the charge and the phosphate additive, while the CaCO_3_ charge increased the time before the flame was extinguished, inhibiting the activity of flame additives^25^. Similar to the result observed in the present work, the authors state that pure HDPE cannot be classified because the flame is not extinguished alone.

Beltrán-Ramírez et al.^27^ investigated the activity of aluminum and magnesium hydroxide particles as flame retardants. The authors noted that several metal hydroxides act as flame retardants and demonstrate that reducing particle size and combining more than one metal hydroxide dramatically reduces the time for flame extinguishing. The mining waste used to produce the E1 composite has a mineral filler consisting of a mixture of many hydroxides and metal oxides, as seen in EDS. The presence of these components may justify the modification promoted to the composite, promoting the delay of the burning of the material.

However, it can be stated that the presence of iron mine tailing in the composite promotes burn retarding activity, increasing the number of possibilities of application of this waste in high added value materials. The composite HPDE/iron mine tailing can be used in a composition of wood plastic composites. According to Krehula et al.^26^, the ammonium polyphosphate (APP) is known to be an effective flame retardant to plastic wood samples and fit into V-0 classification according to a UL 94 test. However, the APP generated a drop in tensile strength of HDPE and its degradation takes place earlier and promotes char formation.

In the work of Beltrán-Ramírez et al.^27^, the authors showed that the specific compositions of the composites valuated with HDPE/particles aluminum and magnesium hydroxide fit into V-0 classification and other V-1 according to a UL 94 test. Furthermore, there was necessary the addition of compatibilizer to improve the tensile stress. On the other hand, in this present work, it is possible to verify that the composite HPDE/iron mine tailing fit into V-0 classification according to a UL 94 test, the filler improved the tensile stress and maintenance of thermal stability of HPDE.

## Conclusion

In this work, four composites based on HDPE and iron mine tailing, in the proportion 80/20 %wt were produced by different methodologies. Independently of the methodology, it was possible to produce the composites with an enhancement of the properties evaluated in relation of HDPE. However, the use of batch mixer is interesting in the production of composites based on HDPE/iron mine tailing. Since it presented less loss of fillers during processing and a better dispersion of them. The analysis of FTIR spectra to evaluate the proportion of iron mine tailing present in the composite have shown effectiveness.

The results have shown that iron mine tailing can act as a functional filler to HDPE because it improves the properties of the polymer. The iron mine tailing acted as a reinforcement agent, enhancing the mechanical strengths, about 40% of Young's modulus and 11% of tensile strength limit when compared to the properties of pure polymer, and an efficient flame-retardant activity in E1 samples. The replacement of HDPE pure by the composite HDPE/iron mine tailing in several uses present an alternative to the disposal of iron mine tailings.
